# Perineuronal Nets and Their Role in Synaptic Homeostasis

**DOI:** 10.3390/ijms20174108

**Published:** 2019-08-22

**Authors:** Mateusz Bosiacki, Magdalena Gąssowska-Dobrowolska, Klaudyna Kojder, Marta Fabiańska, Dariusz Jeżewski, Izabela Gutowska, Anna Lubkowska

**Affiliations:** 1 Department of Functional Diagnostics and Physical Medicine, Pomeranian Medical University in Szczecin, Żołnierska 54 Str., 71-210 Szczecin, Poland; 2 Department of Cellular Signalling, Mossakowski Medical Research Centre, Polish Academy of Sciences, Pawińskiego 5 Str., 02-106 Warsaw, Poland; 3 Department of Anaesthesiology and Intensive Care, Pomeranian Medical University in Szczecin, 71-252 Szczecin, Poland; 4 Institute of Philosophy, University of Szczecin, Krakowska 71-79 Str., 71-017 Szczecin, Poland; 5 Department of Neurosurgery and Pediatric Neurosurgery, Department of Applied Neurocognitivistics, Pomeranian Medical University in Szczecin, 71-252 Szczecin, Poland; 6 Department of Human Nutrition and Metabolomics, Department of Medical Chemistry, Pomeranian Medical University in Szczecin, Broniewskiego 24 Str., 71-252 Szczecin, Poland

**Keywords:** perineuronal nets (PNNs), extracellular matrix (ECM), synaptogenesis, neuronal communication

## Abstract

Extracellular matrix (ECM) molecules that are released by neurons and glial cells form perineuronal nets (PNNs) and modulate many neuronal and glial functions. PNNs, whose structure is still not known in detail, surround cell bodies and dendrites, which leaves free space for synapses to come into contact. A reduction in the expression of many neuronal ECM components adversely affects processes that are associated with synaptic plasticity, learning, and memory. At the same time, increased ECM activity, e.g., as a result of astrogliosis following brain damage or in neuroinflammation, can also have harmful consequences. The therapeutic use of enzymes to attenuate elevated neuronal ECM expression after injury or in Alzheimer’s disease has proven to be beneficial by promoting axon growth and increasing synaptic plasticity. Yet, severe impairment of ECM function can also lead to neurodegeneration. Thus, it appears that to ensure healthy neuronal function a delicate balance of ECM components must be maintained. In this paper we review the structure of PNNs and their components, such as hyaluronan, proteoglycans, core proteins, chondroitin sulphate proteoglycans, tenascins, and Hapln proteins. We also characterize the role of ECM in the functioning of the blood-brain barrier, neuronal communication, as well as the participation of PNNs in synaptic plasticity and some clinical aspects of perineuronal net impairment. Furthermore, we discuss the participation of PNNs in brain signaling. Understanding the molecular foundations of the ways that PNNs participate in brain signaling and synaptic plasticity, as well as how they change in physiological and pathological conditions, may help in the development of new therapies for many degenerative and inflammatory diseases of the brain.

## 1. Introduction

Approximately 10–20% of the brain volume is occupied by the extracellular matrix (ECM), which is a dense network of proteins and glycans that provides anchorage points for nerve and glial cells, contributes to their normal physiology [1,2], and it is crucial for communication between neurons and glial cells, as well as synaptic plasticity. Three ECM compartments can be distinguished: the basement membrane, the interstitial matrix, and perineuronal nets (PNNs) [3]. The basement membrane forms a barrier between the endothelium and the parenchyma. The neural interstitial matrix is located in the brain parenchyma, while PNNs are found around neurons. When compared to similar structures found in other tissues of the body, brain ECM has higher concentrations of hyaluronic acid (hyaluronan, HA), thrombospondin, and proteoglycans, such as: neurocan, aggrecan, phosphacan, versican, brevican, and tenascin [4]. Other important structural and functional components of the brain ECM include fibronectin, elastin, and entactin [5]. The ECM components serve as ligands for cellular adhesion receptors (integrins), through which they transmit signals to the cell’s interior [6]. The synaptic cleft and the space surrounding synapses contain a specialized form of ECM, which is likely dominated by tenascin, thrombospondin, and neuronal pentraxins [7]. Both physiological (cognitive) and pathological (neurodegenerative) processes are accompanied by specific ECM proteolysis events that are catalyzed by metalloproteinases (MMPs) and serine proteases [8].

PNNs surround cell bodies and dendrites, leaving free space for synapses to come into contact [9]. Their structure is still not known in detail, especially given that they lack a defined composition and rather form highly heterogeneous structures, although they were discovered more than a hundred years ago by Camillo Golgi and were first described in 1999 by Spreafico et al. [10]. The best-known histological technique to detect the presence of PNNs is lectin-agglutininin isolated from *Wisteria floribunda* (WFA), which binds to N-acetylgalactosamine in the polysaccharide chain of most PNNs. Histological staining with WFA has revealed the presence of PNNs throughout the entire brain, especially around fast-spiking parvalbumin interneurons (PV cells) [11]. The functional testing of PNNs utilizes chondroitinase ABC (ChABC), an enzyme that degrades chondroitin sulfate into disaccharides. This enzyme can be fed directly into the brain of experimental rats, and its action aids in understanding the importance of PNNs not only for PV cell activity, but also for learning, memory trace formation, and neurodegeneration (see Lau et al. 2013, [3]). The functions of individual ECM components have been studied in detail by enzymatic digestion of glycans, as well as by the use of knockout mouse models, antibodies, and recombinant proteins.

In this paper, we review PNN structure and components, including HA, proteoglycans, core proteins, chondroitin sulphate proteoglycans, tenascins, and Hapln proteins. We also characterize the role of ECM in the functioning of the blood-brain barrier (BBB), neuronal communication, as well as the participation of PNNs in synaptic plasticity and some clinical aspects of PNN impairment. In recent years, many papers have been published, including reviews (see e.g., Sorg et al. 2016, [12], Bozzelli et al. 2018, [13], and Reicheft et al. 2019, [14]), in which the authors present a conception of brain plasticity that is based on cooperation, not only between neurons and glial cells, but also including PNNs. We must consider the role of PNNs in brain signaling in order to properly understand the normal physiological functioning of the brain, e.g., learning and memory, as well as the pathological processes underlying brain disorders. In our review, we discuss the participation of PNNs in signaling pathways in the brain. Understanding the molecular foundations of the ways that PNNs participate in brain signaling and synaptic plasticity, as well as how they change in physiological and pathological conditions, may help in the development of new therapies for many degenerative and inflammatory diseases of the brain.

## 2. The Role of Extracellular Matrix in the Functioning of the Blood-Brain Barrier

In addition to its structural functions, the ECM is involved in supplying metabolites from the peripheral circulation to neurons and glial cells, and in removing the metabolic products of these cells back into the bloodstream. The proper functioning of the BBB is especially important for this transport function, which, due to its selective permeability, acts as a dynamic link between the peripheral circulation and the brain [15,16]. Its structural and functional integrity is essential for maintaining quantitative and qualitative homeostasis of the brain. The barrier consists of a complex structure of brain microvascular endothelial cells (BMECs) surrounded by astrocytes and pericytes, which, together, create and maintain the structure of microvessels in the brain [10]. The strength of the capillaries forming the BBB is increased by the presence of tight junctions (TJs) [17], intricate protein complexes mediating adhesion between cells, and the regulating transport through the cell membrane. Together, the BBB and ECM form an efficient medium for molecular exchange between the peripheral circulation and central nervous system [18]. In addition, the ECM is crucial for the proper functioning of the BBB. For example, by increasing the expression and activity of relevant MMPs, the ECM affects the remodeling of TJs and other ECM proteins, which directly affects the properties of the BBB [18,19].

## 3. Structure of Perineuronal Nets

The cerebral ECM consists of many different glycosaminoglycans (GAGs), proteoglycans (PGs), and proteins that condense around certain neurons to form PNNs. PNN components are synthesized by neurons, astrocytes, and oligodendrocytes, forming a unique microenvironment. The basic components of PNNs are: (1) HA the main structural component in terms of volume, and number of enzymes catalysing its synthesis, (2) chondroitin sulphate proteoglycans (CSPGs) including aggrecan, brevican, neurocan, and versican; the main proteoglycans forming PNNs, (3) tenascin; in particular Tn-R, and (4) hyaluronan and proteoglycan binding link protein (HAPLN-1, -3 and -4) [3]. 

PNNs surround the perikaryon and proximal dendrites of nerve cells. Numerous components cooperate to form a condensed network, which is additionally modulated by MMPs that locally degrade PGs. Polysaccharides of GAGs that are found in PNNs have different sequences consisting of two-saccharide subunits. Further complexity of the structure arises from the modification of polysaccharides in the ECM [3], Table 1 and Table 2.

## 4. Characteristics of Perineuronal Nets Components

### 4.1. Hyaluronan 

Hyaluronan (HA) is a polymer that is comprised of hundreds of repeated disaccharide subunits: glucuronic acid-acetylgalactosamine (GlcA-GlcNAc) and it is the only glycosaminoglycan in PNNs that is not sulfated nor bound to the protein core [20]. HA is directly formed in the extracellular space by the transdermal enzyme hyaluronan synthase (HAS). There are three HAS isoforms (HAS1, HAS2, HAS3). HAS3 synthesizes HA more slowly and produces shorter polymers (∼105 Da) than HAS1 and HAS2 (∼106 Da) [21]. The expression of isoforms can differ according to the area of the brain, e.g., all of the isoforms are expressed in hippocampal neurons, whereas cerebellar neurons express only HAS2 and HAS3 [22]. Additionally, all three isoforms are expressed in the perikaryon, whereas only HAS1 was found in axonal cell membranes [23]. 

HA is the main component of PNNs, providing the primary skeleton to which PGs are attached. An in vitro study showed that HAS3–transfected HEK cells are capable of condensing the ECM and forming structures that are similar to PNNs [24]. Due to their length, HA polymers can bind up to one hundred proteins simultaneously, and since the polymers have different lengths, the ECM structure is heterogeneous and exhibits different mechanical properties, depending on the location. HA is responsible for PNN hydration, and therefore ECM viscosity. Although HA can bind different molecules and signaling proteins (e.g., CD44), this has not yet been observed in PNNs [22]. 

### 4.2. Proteoglycans

#### 4.2.1. Glycosaminoglycans 

Proteoglycans (PGs) constitute the protein core to which glycosaminoglycans (GAGs) are attached though a bond between the tetrasaccharide xylose in GAGs and a protein core serine, catalyzed by β-D-xylosyltransferase. Every GAG consists of repeating disaccharide subunits, each of which contains an amino-sugar—a derivative of galactose (GalNAc N-acetylogalactosamine) or glucose (GlcNAc N-acetyloglucosamine)—connected to galactose (Gal) or a uronic acid (glucuronic acid, glucosamine (GlcA) or iduronic acid (IdoA)). Chondroitin sulphate (CS) is a GAG which consists of repeated units of GlcA and N-acetylogalactosamine (GalNAc); dermatan sulphate (DS) consists of IdoA and GalNAc; heparan sulphate (HS) consists of GlcA or IdoA and GlcNAc; and, keratan sulphate (KS) consists of Gal and GlcNAc. 

Many different enzymes synthesized homogeneous disaccharide polymers (GAG-GAG). For example, the assembly of CS and HS polymers begins with the synthesis of a tetrasaccharide linking region through the sequential addition of monosaccharides (xylulose, galactose, galactose, and glucuronate) by the corresponding glycosyltransferase enzymes (xylosyltransferase, GalT-I, GalT-II) glucuronyltransferase (GlcAT-1), respectively) [25,26]. The enzyme (3)β-D-xylosyltransferase connects GAGs, through the tetrasaccharide linking region, to the protein core serine [26]. Polymer elongation (CS chains) is catalyzed by transferases GalNAcT-II and GlcAT-II, which add GalNAc and GlcA, respectively. Proteoglycan variability stems from the number of repeated disaccharide fragments, which range from 20 to 200, and the ratio between various GAGs. In the human CNS, the HS:CS ratio is 9:1, but CS is more abundant in PNNs [25].

#### 4.2.2. Core Proteins

The core proteins of chondroitin sulphate proteoglycans (CSPGs) consist of two domains: the global G3 at the C-terminus and one or two global domains (G1 and G2) at the N-terminus. These domains bind to proteins that participate in the condensation of the ECM into PNNs. The G1 domain contains an immunoglobulin module and two repeating PGS. The G2 domain is only found in aggrecan and it contains two repeating PGS. Repeating proteoglycans in the G1 domain mediate the binding of HA by interacting with Hapln family proteins [27]. The G3 domain contains an EGF fragment, lecithin domain, and complement regulatory protein (CRP) [25]. Calcium ion-dependent binding of the lecithin domain with tenascin-R (Tn-R) and tenascin-C (Tn-C) contributes to the condensation of the ECM (i.e., PNN formation) [28,29,30].

#### 4.2.3. Chondroitin Sulphate Proteoglycans 

CSPGs in the brain can be divided into three groups: (1) secretory CSPGs, (2) membrane CSPGs, and (3) glycosylphosphatidylinositol-anchored CSPGs. It is also possible to distinguish the family of hyaluronan-binding PGS, called lecticans, which consists of versican, aggrecan, brevican, and neurocan [31], all of which are secretory PGS, except for the GPI-anchored brevican [28,31,32]. Lecticans have a common structure of their N-terminal domain, which binds HA, and their C-terminal domain, which binds lectin, as well as a characteristic structure in the middle, which contains GAGs. PGS not only play a structural role in the brain, but they are also involved in its development by interacting with growth factors and molecules regulating cell adhesion [31,32]. Through chondroitin sulphate chains and core proteins, they can inhibit neurite growth and regeneration [20]. 

In brain injury, proteoglycan expression increases [33,34,35] and GAG chains are enzymatically removed by chondroitinase, which promotes axon regeneration and neuronal restoration after injury [36]. The reconstruction of CSPGs is observed in many neuropathological and neurodegenerative diseases of the central nervous system, such as schizophrenia, Alzheimer’s disease (AD), perivascular dementia, and epilepsy, which may indicate a role for the ECM in numerous pathomechanisms in the brain [37].

#### 4.2.4. Tenascins

The tenascin family consists of four different glycoproteins, but only Tn-R and Tn-C have been observed in the brain thus far, in neurons and glial cells [23]. Tenascins are highly heterogeneous due to changes during posttranslational processing and the activity of MMPs. Tenascin-C has a quaternary structure that consists of two homotrimers, thus forming a hexamer. Tn-R forms both homodimers and homotrimers. Tn-R associates with the lectin domain of CSPGs via its fibrinonectin type III domain, which repeats 3–5 times and it is crucial for PNN structure [38,39].

#### 4.2.5. Hapln Proteins

Hapln proteins form a non-covalent connection between HA and the G1 lectin domain of PGS, preventing the diffusion of lectins into the ECM. The Hapln family consists of four different proteins, three of which have been detected in the brain: Hapln1 (Crtl1), Hapln2 (brain-specific hyaluronan binding protein (Bral1)), and Hapln4 (Bral2). Hapln1 and Hapln4 are expressed in neurons that wee surrounded by PNNs, while Hapln2 is expressed in oligodendrocytes and it is also present in PNNs surrounding nodes of Ranvier that were assembled by versican V2 [27]. In the brain, Hapln1 binds to all lectins and Hapln4 with brevican, forming a complex with CSPGs and HA. A study by Kwok et al. 2010 [24] investigating the mouse cell cultures with HAS3 and Hapln1 (Crtl1) overexpression showed the essential role of Hapln for the formation of PNNs. Similar results were observed in a study on Hapln1 knockout mouse cell cultures [22].

## 5. Neuronal Communication

Communication between neurons defines brain function. It occurs through intact synapses that are able to transmit and receive chemical signals. The basic medium of neural communication, synaptic transmission, occurs primarily at chemical synapses, which are subject to rapid and effective neurotransmitter secretion [40,41]. The task of the axon is to transfer the membrane potential to the other cell, which is achieved when the change in potential triggers the fusion of synaptic vesicles (SVs) that are filled with neurotransmitters with the presynaptic membrane. The neurotransmitter that is released into the synaptic cleft is then bound by its respective receptor located in the active part of the dendrite membrane, thus releasing a new wave of action potential in the postsynaptic cell [42]. This process forms the basis of intercellular communication in the nervous system and it is crucial for memory and learning [43].

**Figure 1:**
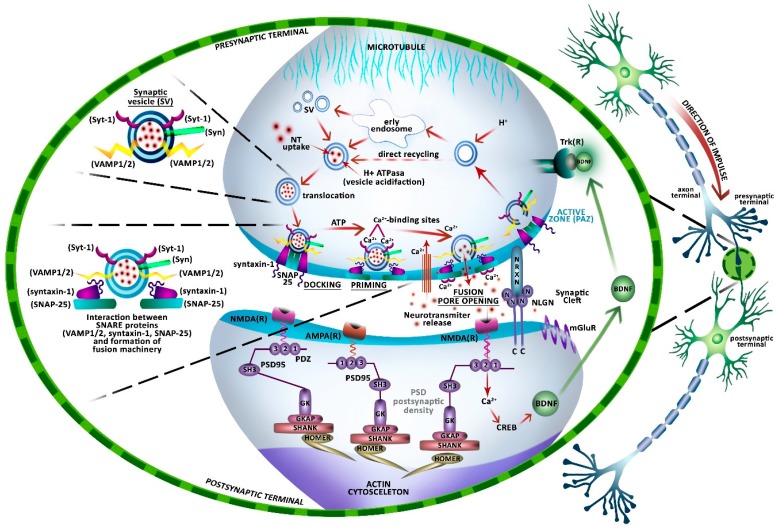
Schematic diagram showing neuronal communication. Neurotransmitter (NT; red dots) release is mediated by exocytosis of synaptic vesicles (SVs) (blue circles) at the presynaptic active zone (PAZ) of nerve terminals. SVs are filled with neurotransmitters (neurotransmitter uptake) by active transport fuelled by an electrochemical gradient established by a proton pump (H^+^-ATPase) that acidifies the vesicle interior. The action potential, conducted orthodromically along the axon, opens voltage-gated Ca^2+^ channels in the membrane of the presynaptic area, near the PAZ, results in an increase in Ca^2+^ concentration and in the migration and anchoring/docking of SVs to the presynaptic membrane in the PAZ. Docked SVs go through a maturation process called priming (ATP-dependent process of SVs activation/priming renders the SVs competent to a Ca^2+^ signal and fuse with the plasma membrane). Interactions between different proteins at the PAZ mediate attachment of the SVs to the target membrane. VAMP proteins, also called synaptobrevins (VAMP1/2), belonging to the class of proteins found in vesicular membranes, i.e., v-SNAREs bind to SNAP25 (synaptosomal nerve-associated protein, weighing approximately 25 kDa) and anchor protein syntaxin-1, presynaptic plasma membrane proteins (t-SNARE), forming the fusion machinery complex (SNARE complex). When an action potential depolarizes the presynaptic membrane and opens voltage-gated Ca^2+^ channels (calcium signalling), local increase in intracellular Ca^2+^ level in PAZ triggers fusion reaction (fusion pore opening) by binding to Ca^2+^ sensor protein: synaptotagmin-1 (Syt-1). Full SNARE-complex assembly then pulls the membranes apart, opening the fusion pore, which expands such that the vesicle membrane collapses into the target membrane. After fusion pore opening, SVs are re-endocytosed, recycled and refilled with neurotransmitters. Neurotransmitters present in SVs are released to synaptic cleft and are bind to the receptors (R) associated with the postsynaptic membrane. Neurotransmitter receptors (glutamate receptors, such as: N-methyl-D-aspartate receptors (NMDA(R)), alpha-amino-3-hydroxy-5-methyl-4-isoxazole propionic acid receptors (AMPA(R)) and metabotropic glutamate receptor (mGluR)) together with cytoskeletal proteins are clustered in the postsynaptic density through the scaffolding proteins. The PDZ-domain-containing scaffold postsynaptic density protein-95 (PSD95, also known as DLG4), Src-homology domain 3 (SH3), guanylate kinase-like domain (GK) and multiple ankyrin repeat domains (SHANK) family proteins form a protein network below the postsynaptic membrane, which is bridged by guanylate kinase-associated protein (GKAP). Numbered circles indicate the first, second, and third PDZ domains of PSD-95. The PSD is a very crowded region, and only a select number of the full complements of molecules have been included. Presynaptic and postsynaptic membranes are connected by cell-adhesion molecules (Neurexins (NRXN) and Neuroligins (NLGN)). The production and release of BDNF depends on the postsynaptic NMDA receptor activation, as a result of the fusion of the SVs and release of glutamate. The NMDAR-dependent release of BDNF may be critical in the creation of sites of presynaptic liberation of neurotransmitters. Abbreviation’s: Homer protein (HOMER), Tropomyosin receptor kinase B (Trk(R)), cAMP response element binding protein (CREB).

Specialized proteins that are found in SVs and cell membranes mediate neurotransmission (Figure 1). Neurotransmitters are taken up and stored in presynaptic neuron SVs by vesicular transporters, ion channels, and vesicular H^+^-ATPase, which supplies the transporters with the necessary proton gradient [44,45]. The action potential, conducted orthodromically along the axon, opens the voltage-gated calcium channels in the membrane of the presynaptic cell, near the presynaptic active zone (PAZ). This results in an increase in calcium (Ca^2+^) concentration and in the migration and anchoring of SVs to the presynaptic membrane in the PAZ [46]. Synapsin is a phosphoprotein that regulates the number of SVs that are available for exocytosis. It is involved in tethering SVs to the cytoskeleton away from the PAZ and only its phosphorylation during an action potential induces the release of SVs from the reserve pool, allowing for their movement toward the active zone and the release of neurotransmitters [46].

SVs docking, which is one of the first stages of exocytosis in neurons and neurosecretory cells, depends on the formation of the molecular machinery that is responsible for the fusion of secretory vesicles with the cell membrane, i.e., the 7S SNARE complex (soluble N-ethylmaleimide sensitive factor attachment protein (SNAP) receptor) [47,48,49,50]. SNARE complexes, which are present at all types of synapses, consist of three SNARE proteins, taking their name from their roles as receptors for SNAP proteins. These are: VAMP proteins, also called synaptobrevins, which belong to the class of proteins found in vesicular membranes, i.e., v-SNAREs, and two specific proteins associated with the target membrane, i.e., t-SNAREs—anchor protein syntaxin-1 and cytoplasmic SNAP-25 (synaptosomal nerve-associated protein, weighing approximately 25 kDa), which is bound to the membrane by palmitoylation of cysteine residues [47,50,51].

The VAMP family consists of seven proteins that are involved in vesicle fusion: synaptobrevin 1 (VAMP1), synaptpbrevin 2 (VAMP2), cellubrevin (VAMP3), VAMP4, myobrevin (VAMP5), Ti-VAMP (VAMP7), and endobrevin (VAMP8) [52]. They are 13 to 32 kDa membrane-associated proteins that are largely exposed to the cytoplasmic side of the SVs membrane [53]. VAMP1 and 2 are expressed in the SVs of neurons and in the secretory granules of endocrine and exocrine cells [49]. VAMP binds to the SNAP-25–syntaxin-1 heterodimer, forming a 7S complex, which is a SNAP receptor [44,48]. It has been proposed that the association of VAMP with syntaxin-1, possibly due to the dissociation of synaptophysin (Syp), a glycoprotein associated with vesicles and attached to VAMP, initiates the formation of fusion pores [44,48]. However, this mechanism is not well understood and there are also studies that have refuted this proposed role for Syp [54]. The created complexes of v-SNARE and t-SNARE proteins act as binding sites for other proteins that are involved in membrane fusion. According to the SNARE hypothesis—that describes the molecular mechanism of secretory vesicle fusion with the acceptor membrane as the interaction of specific proteins located in the membranes of these two compartments—the next stages of exocytosis are the activation/priming and fusion of SVs with the plasma membrane [53]. Anchored in the membrane of the PAZ, SVs are primed in an ATP-dependent process, poised for fusion in response to a Ca^2+^ signal. An increase in intracellular Ca^2+^ level, which is caused by depolarization of the presynaptic membrane and the opening of voltage-gated Ca^2+^ channels, triggers exocytosis (fusion pore opening) through the binding of Ca^2+^ ions to the calcium-sensor protein: synaptotagmin-1 (Syt-1). Synaptotagmin, a 65 kDa glycoprotein, is found in synaptic vesicle membranes. It contains two C2 calcium-binding motifs, which are located on the cytoplasmic side of the membrane, which facilitate the cooperative binding of phospholipids in a Ca^2+^-dependent manner [55,56]. There are 15 known isoforms of this protein with varying Ca^2+^ affinities. Syt-1 is necessary for the Ca^2+^-dependent process of neurotransmitter secretion. Neurotransmitters that are released into the synaptic cleft as a result of exocytosis bind to receptors in the postsynaptic membrane [50,51,52,53,54,55,56,57,58,59].

In the neocortex and hippocampus, most of the axodendritic synapses are located on dendritic spines, tiny protrusions from the dendritic trunk that increase the density of synaptic connections in nervous tissue [60]. In electron microscope images, synapses on dendritic spines are characterized by the presence of postsynaptic densities (PSD), electron-dense, and amorphous material adhering to the postsynaptic membrane. The PSD contains proteins that connect the cell membrane with the cytoskeleton, many signaling proteins involved in neurotransmission and associated with postsynaptic membrane receptors, as well as receptors for neurotransmitters [61]. The PSD includes several hundred proteins that are capable of forming large, heteromeric, and dynamically changing protein complexes [62,63]. The structure ensures efficient signal transmission, namely through so-called scaffold proteins. These proteins, through interactions and connections with other proteins, form a complex spatial network that organizes and localizes proteins at the postsynaptic membrane into appropriate signalling paths [61]. In the PSD, we distinguish three main families of scaffold proteins: membrane-associated guanylate guanyl kinases (MAGUK), SH3 and multiple ankyrin repeat domain (SHANK) proteins, and Homer [64,65,66]. The dominant scaffold protein in PSD, which plays the most important role in the formation of the entire PSD structure, is PSD-95 (the PSD protein with molecular weight 95 kDa, also known as synapse-associated protein 90, SAP-90). It is estimated that the amount of PSD-95 is ten times greater than the amount of ionotropic N-methyl-D-aspartate (NMDA) receptors and seven times greater than the amount of Shank proteins [67,68]. PSD-95 belongs to the family of MAGUK proteins and it is present in large amounts at glutamatergic synapses, forming complexes with the NMDA receptor. This protein increases the number and size of the dendritic spines, thus regulating NMDA-receptor-dependent changes in the number of α-amino-3-hydroxy-5-methyl-4-isoxazolepropionic acid receptors (AMPARs), and contributes to synapse stabilization and plasticity [69,70].

Synapses with PSD are generally excitatory synapses; glutamate acts as a neurotransmitter and binds to postsynaptic ionotropic NMDA, AMPA, and kainate receptors, or to a metabotropic receptor [60,71]. Synapses that do not have visible PSD in the electron image usually contain the inhibitory neurotransmitter—gamma-aminobutyric acid (GABA). Inhibitory synapses are mainly formed on the shaft of dendrites or around the neuronal cell body, and by electron microscopy only show a slight electron-dense thickening that is associated with the postsynaptic membrane. Hence, they were described as symmetric (type II) synapses. This presumably reflects the fact that inhibitory postsynaptic specialization is much less elaborate than the PSD of excitatory synapses [72].

Synapses are surrounded by astrocytes that provide metabolic components, contribute to the antioxidant protection of neurons, retrieve neurotransmitter from the synaptic cleft, and participate in synaptic transmission and synaptogenesis [73].

In the developing brain, the stabilization of presynaptic neurotransmitter release sites is regulated by the postsynaptic feedback signals [74,75]. One such signal is brain-derived neurotrophic factor (BDNF), which belongs to the group of secretory polypeptides, the so-called neurotrophins. Together with other proteins, such as nerve growth factor (NGF), neurotrophins NT-3, NT-4/5, and NT-6 (synapse-supporting proteins), and BDNF takes part in neuronal function and affects the central and peripheral nervous system [76]. Being teleased in a process that is dependent on NMDA receptor activation, BDNF binds to tropomyosin receptor kinase B (TrkB). TrkB activation in the presynaptic neuron increases the number of docked SVs and the incorporation of Syp and VAMP1/2 into SVs, thus stimulating and intensifying the process of synaptic exocytosis [74]. NMDAR-dependent BDNF release seems to play a critical role in the creation of presynaptic neuromediator release sites [77,78]. BDNF regulates the survival and growth processes of nerve cells and it affects neuronal ultrastructure, axon morphology, and synaptic connections. It also acts as a modulator of neurotransmission, induces changes in the expression of pre- and postsynaptic proteins, and participates in neuronal plasticity, crucial for learning and memory [67,79,80]. Research shows that the highest concentrations of BDNF are found in the hippocampus, amygdala, neocortex, and cerebellum [80,81].

There is extensive data indicating that disturbances in proteins that are involved in maintaining proper synapse structure, distribution, release of SVs, as well as in synaptic transmission (e.g., VAMP1/2, Syntaxin-1, SNAP-25, Syp, Syt-1, PSD-95, and other scaffold proteins of the MAGUK or Shank families) are common molecular mechanisms underlying synaptic pathology, neuronal dysfunction, and cognitive disorders that are observed in both neurodegenerative and neurodevelopmental diseases, i.e., autism spectrum disorders (ASD), schizophrenia, and AD [82,83,84]. Changes in the concentration and activity of neurotrophins, especially BDNF, are also of key importance in the pathogenesis of neurodegenerative diseases (AD, Parkinson’s disease (PD), Huntington’s disease, dementia) and neurodevelopmental disorders (ASD) [85]. BDNF deficiency is observed in neurons that are rich in neurofibrillary tangles (NFTs) in AD, while disorders in its synthesis are associated with mutations of α-synuclein protein (ASN) in the hereditary form of early onset PD [86,87,88]. A decrease in BDNF concentration is also observed in schizophrenia, which suggests the involvement of this factor in the disease etiology [89]. It was found that synapse degeneration is the main manifestation of pathology observed in various forms of dementia, especially in AD [90]. There is also evidence to support the important role of synaptic protein-coding genes in the pathophysiology of ASD. The identification of mutations in numerous genes encoding proteins important for synapse formation, maturation, and stabilization (i.e., presynaptic neurexins (NRXN1, NRXN2, NRXN3), their postsynaptic neuroligin ligands (NLGN1, NLGN3, NLGN4X, NLGN4Y), as well as the family of SHANK proteins found in the PSD (SHANK1, SHANK2, SHANK3)) have indicated synapses as the central players in determining ASD susceptibility [91,92,93,94,95].

## 6. Participation of Perineuronal Nets in Synaptic Plasticity

Synaptic plasticity refers to changes in synaptic structure and transmission strength during brain development and in the mature brain. Plasticity allows for synapses to meet the specific functional and adaptive requirements of a changing environment. Synapse activation may have both short and long term effects, with the latter being associated with a permanent increase in the strength of synaptic transmission [8]. Long-term plasticity involves intracellular signalling cascades and the regulation of gene expression.

Underlying plasticity are processes of structural and functional modification. The former include changes in the shape, number, and size of previously formed synapses and the creation of new connections [42]. Functional changes involve modification to the number and activity of neurotransmitter receptors in the postsynaptic and presynaptic cells, as well as changes in the intensity of neurotransmitter secretion. Long-term potentiation (LTP) and long-term depression (LTD) of synaptic transmission are now widely recognized as representing cellular mechanisms that are used by neuronal networks to store certain types of information. The molecular and cellular events that underlie these forms of activity-dependent synaptic plasticity are also being elucidated [96,97].

An important factor that influences synaptic plasticity is the specific synthesis and proteolysis of ECM components [96].

In the mature brain, PNNs play a key role in synaptic plasticity by creating a physical barrier to control the changes in the formation of new connections between neurons, allowing for the axon of one neuron to connect with the body of another neuron or preventing the formation of a connection between neurons [97]. PNNs also act as scaffolding for synaptic connection inhibitors (e.g., semaphorin 3A), which prevent the formation of new neuronal links. PNNs have also been shown to limit the mobility of ionotropic AMPARs on the surface of neurons, which hinders the exchange of desynthesized synapse receptors for new receptors on the extrasynaptic side [98].

PNNs also play a key role in the developing brain, as evidenced by the high levels of expression of matrix components that were observed before the peak of postnatal synaptogenesis, which suggests the importance of PNNs for the formation of synapses in the immature, developing brain [99]. The results of in vitro studies on primary neuronal cultures have shown the expression of aggrecan on the surface of neurons, which may determine the location of future synapses [100].

PNNs are undoubtedly important in memory processes [101]. In adult mice, fear conditioning results in the storage of long-term extinction-resistant memory traces. The extinction of fear-conditioned memories during early postnatal development suggests that such memories are actively protected in adult mice. A study found that this protection was provided by increasing the expression of CSPGs, which are a component of PNNs, in the amygdala. It was also observed that the organization of CSPGs into PNNs coincides with the switch from erasure to protection of fear-conditioned memories. These results indicate that PNNs mediate the formation of extinction-resistant fear-conditioned memories and they may be instrumental in the identification of a molecular mechanism that closes the critical period in early neonatal development during which traumatic memories can be erased [101]. Changes in physiological states, such as wound healing, gliosis, aging, and psychiatric and neurological disorders, alter the expression of ECM components [102], which suggests that ECM plasticity still exists after brain maturation and it may be an important aspect of pathophysiological changes in the CNS.

Reactive astrocytes exhibiting high expression of glial fibrillary acidic protein (GFAP) are functionally and morphologically distinct from non-reactive astrocytes and they are responsible for preventing injury progression in damaged brain regions by participating in the formation of a glial scar (gliosis). The accumulation of reactive astrocytes in the brain is also associated with the normal aging process, as well as with the formation of amyloid β (Aβ) plaques in AD and glial scars in brain injuries [103].

Neurotoxins, cytokines, and inhibitory ECM molecules that are secreted by reactive astrocytes constitute a chemical barrier around damaged neuronal populations [104]. Among ECM molecules, the up-regulation of tenascin proteins (Tn-C/Tn-R), HA, and some CSPGs has been observed, and they are responsible for limiting axonal elongation of the glial scar [105,106]. The increased expression of CSPG has been observed in various trauma cases. Intensified expression of neurocan and phosphacan was observed in reactive astrocytes in the vicinity of the scar following cerebral cortex injury, while no expression of brevican and versican was observed [107]. It was also demonstrated that antibody blocking of PGS as well as enzymatic modification with either ChABC or heparinase increased the growth of neurites above the surface of the glial scar in the adult rat cortex in vitro [108] and in vivo, respectively [107].

PNNs have also been shown to contribute to neurodegenerative diseases, such as AD [109]. For example, the increased expression of brevican in the frontal gyrus was observed in patients with AD. Recent in vitro studies have shown that the brevican core protein binds oligomeric and fibrillar Aβ1-42 peptides, but not the monomeric form [110]. The injection of ChABC into the hippocampus of AD mice reduced the loss of postsynaptic density around Aβ plaques. In vivo treatment transgenic APP/PS1 mice with ChABC alleviated AD symptoms, such as the loss of hippocampus-dependent short-term memory and LTP impairment. In addition, the accumulation of CSPGs, especially brevican, in stimulated astrocytes, impairs the synaptic functions in AD [110].

## 7. Perineuronal Nets - Clinical Aspects

In nervous system pathophysiology, swelling, which increases the distance between components of the perineuronal space, and bleeding are invariable elements. PNNs can take on either the role of activator [111] or the inhibitor of pathological processes [112], as they are a space in which nerve and glial cells interact, and thus mediate ionic communication between them [113]. Bonneh-Barkay et al. (2009) highlight the role of MMPs and MMP inhibitors (TIMP proteins) in the pathophysiology of neurodegenerative diseases, such as multiple sclerosis and brain tumors [111]. An abnormal ratio of MMPs and inhibitors would, according to the authors, cause an intensification of inflammatory processes. MMPs 1, 2, 3, 7, and 9 are featured in this study and they are found to be upregulated in a variety of neurodegenerative diseases. Concentrations of the MMP inhibitors TIMP1 and TIMP2 also increase in neurodegenerative disease states—which can be paradoxically accompanied by an increase in activity of the MMPs that they control [111]. An additional factor associated with intensifying pathological processes in the brain is increased activation of tPA (tissue plasminogen activator), which may activate microglia, and thus intensify neuropathological processes [111].

Recent studies have shown that PNNs are involved in the pathophysiology of traumatic brain injury (TBI), traumatic spinal cord injury, and stroke [112,114,115,116], as well as in diseases, such as schizophrenia, epilepsy, and autism [112,114,115,117]. TBI is one of the most common causes of death and disability and is thus widely studied [112,114,115]. However, the heterogeneity of this disease causes a lack of scientific consensus on its pathophysiology and treatment.

**Figure 2:**
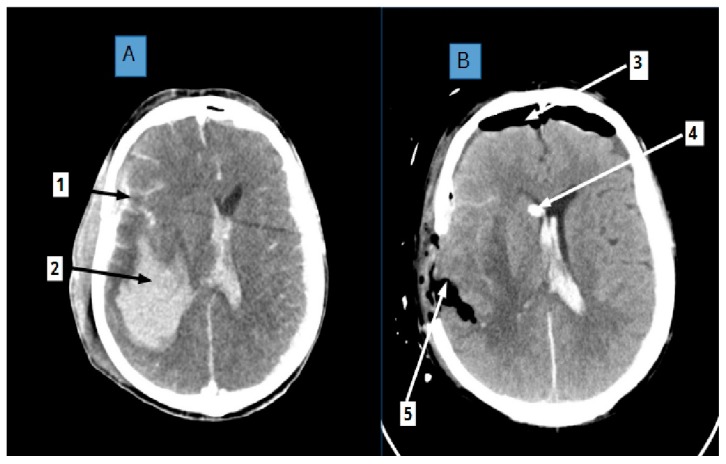
56 years old male, a victim of craniocerebral trauma (CT) due to assault. Assessment upon admission rated him at a 3 GCS (Glasgow Coma Scale) without autonomic respiration. CT **A**: showing huge intracerebral hematoma in right hemisphere (arrow 2) surrounded with edematous area. Subarachnoid hemorrhage (arrow 1) as well as intraventricular blood clot. Right-sided craniectomy was performed and hematoma evacuated. Intraventricular external drainage was installed. CT **B**: shows postoperative cavity with air remnants (arrow 5), intraventricular catheter tip in right frontal horn is also seen (arrow 4). Air in subarachnoid space in frontal region is present due to brain volume reduction (arrow 3). Noteworthy is that the shift of medial line structures is less advanced compared with CT A taken before surgery. The patient survived with the GOS (Glasgow Outcome Scale), which might be related to the early application of decompressive techniques e.g., craniectomy and increased CSF outflow causing more adequate perineuronal net geometry. The study was conducted in accordance with the Declaration of Helsinki, and the Bioethical Commission of Pomeranian Medical University in Szczecin, Poland, approved the protocol (permission numbers KB-0012/156/17).

The heterogeneous clinical picture of brain injury results from metabolic disorders that involve hypoxia and oedematous enlargement of the intercellular space of the brain, as we can see in Figure 2 in a CT scan of a brain after TBI with hematoma and oedema areas. These disorders mutually induce each other and disturb ECM homeostasis (Figure 2).

Intensive care unit (ICU) and brain trauma foundation (BTF) guidelines confirm the benefits of an efficient clearing of the intercellular space through the drainage of cerebrospinal fluid, as shown in Figure 2B and cerebral blood flow in the treatment of TBI, which may lead to ECM normalization, as it indirectly enables the extracellular fluid drainage.

Astrocytes have also been found to play an important role in brain oedema and water balance disturbances following ischemia. Protein channels—aquaporins (AQPs)—are abundant in the feet of astrocytic capillary processes. It has been shown that maintaining stable AQP function limits the escalation of cerebral oedema and post-oedema neurological deficits. Maintaining stable AQP function is also responsible for water passage through AQP channels and increased glutamate, K^+^, lactic acid, free oxygen radicals, and arachidonic acid [118].

As a result of changes in the ECM and the oedema-induced increase in the distance between brain structures, including capillary vessels, astrocytes become swollen, which thus disturbs BBB function.

Hemphill et al. 2015 [112] described the phenomenon of cellular mechanotransduction of physical forces by ECM structures, which may be an important mechanism of injury in TBI. Understanding this phenomenon may enable new potential therapeutic methods [112]. The authors associate these mechanisms with the occurrence of pathologies, such as hemorrhage or edema, but also with more subtle and harder to diagnose ailments, such as diffuse axonal injury (DAI), microvascular damage, and diffuse neuronal injury. Being closely related to the brain parenchyma, these injuries result in increased mortality in the long term (presenting with non-specific symptoms, such as dizziness and deficits in attention, memory, and motor skills). These symptoms disappear with time, but microscopic trauma, not noticeable in routine clinical examination, might persist and result in later neurodegeneration [112]. The authors describe cellular mechanotransduction as the ability of cells to convert mechanical energy into chemical signals—enabling communication between the interior and the exterior of the cell. In the central nervous system, this communication is complicated by the heterogeneity of structures of the cerebral parenchyma, meninges, and vessels, all differing in density and resistance to impact. As shown in computer modeling studies, the ECM is involved in post-traumatic signal transmission via integrin-mediated Rho-associated protein kinase (ROCK) activation [112].

The problems that are associated with the diagnosis of the aforementioned pathologies are related to the manifold nature of the traumatic forces and their varied effects on the brain and its subcellular microcompartments. The importance of cellular mechanotransduction and the ability of cells to convert mechanical phenomena into biological signals have been established for many organs, tissues, and cells in a variety of species. Cell-cell and cell-matrix interactions can influence both physiological and pathological processes, hence their significance in determining the effects of TBI and in remodeling following brain injury [112]. Further research on PNNs gives hope for a deeper understanding of the basics of neural pathophysiology, but also for improving rehabilitation following brain and spinal cord injury. In these processes, the balance between initial inflammation, which is necessary and essential for survival, and chronic inflammation, which may promote permanent neurological dysfunction, seems to be at least partially dependent on PNNs acting as carriers and communicators between individual nervous system cells [116].

Quirico-Santos et al. (2010) [119] illustrated the dual role of PNNs in the pathogenesis of central nervous system disease, who described the role of lecticans in the prevention and improvement of parenchymal tumor invasion [119]. Lecticans are a family of CSPGs containing lectin and HA, which link other structures of the ECM space. As mentioned in the introduction, molecular analysis revealed the existence of four lecticans—aggrecan, versican, neurocan, and brevican. Versican that is produced by glial cells and stem cells in vitro exhibits the ability to limit the excessive outgrowth of nerve cells. Neurocan is present at sites of active neuronal growth, which facilitates the proper maturation of the central nervous system and reduces lesions. On the other hand, the versican isoform that is presented by glial tumor cells, which interacts with the EGFR receptor (epidermal growth factor receptor) and beta-1 integrin, activates an extracellular cascade of kinases, which creates tumor-promoting conditions [119]. Several studies have demonstrated the influence of MMPs and their interactions with the extracellular space and ECM proteins in the pathophysiology of neurological disorders e.g., AD disease and other dementias [120], schizophrenia, epilepsy [121,122,123], hearing disorders, and ASD [115,124,125].

Research on the role of the ECM in age-related neuroplasticity has also produced interesting results. Fox et al. have shown that, in animals, the previously lost neuroplasticity in visual cortex areas is recovered after the administration of chondroitinase ABC, which enables interneural communication by degrading ECM [126].

Perhaps, Camillo Golgi was right, concerning the function, when he described the structures we now know as PNNs as the peripheral reticulum of a nerve cell component on which all nerve cells depend [127].

## 8. Perspectives for Further Research

Many questions arise when discussing perineuronal networks, among which one seems to be of primary importance: how to explain the paradoxical, accelerating, and inhibiting role of PNNs in the pathogenesis of chronic and acute nervous system diseases. Further research certainly must further investigate the relationship between PNNs and cognitive disorders that are mentioned in the review above. An interesting direction for research in surgery and intensive care could be extending studies on mechanotransduction in the continuous monitoring of patients after central nervous system trauma. In combination with samples that were obtained via microdialysis (samples taken in vivo from brain tissue using a special catheter), determining the activity of MMPs would perhaps provide more accurate information for monitoring the patient’s condition and treatment effectiveness. It seems that it would also be interesting to attempt to visualize perineuronal networks in radiological examinations before and after neurosurgical procedures. Furthermore, it may also be reasonable to evaluate the activity of MMPs while using an intraoperative microscope, which may be helpful in setting boundaries, during e.g., tumor resection.

## 9. Summary

ECM molecules that are released by neurons and glial cells form PNNs and modulate many neuronal and glial functions. A reduction in the expression of many neuronal ECM components adversely affects processes that are associated with synaptic plasticity, learning, and memory. At the same time, increased ECM activity, e.g., as a result of astrogliosis following brain damage or in neuroinflammation, can also have harmful consequences. The therapeutic use of enzymes to attenuate elevated neuronal ECM expression after injury or in AD has proven to be beneficial by promoting axon growth and increasing synaptic plasticity. Yet, severe impairment of ECM function can also lead to neurodegeneration. Thus, it appears that to ensure healthy neuronal function a delicate balance of ECM components must be maintained.

Given the extensive effects of ECM alterations, it seems justified to conduct many studies in this field, especially in order to understand the signal pathways that underlie the beneficial and harmful effects of ECM attenuation.

## Figures and Tables

**Table 1:** Sources and location of perineuronal nets (PNNs) components [2]. Neurons, astrocytes, oligodendrocyte, reactive astrocytes provides PNNs build components and interacting proteins: glycosaminoglycans (GAGs), proteoglycans (PGs); linkage proteins; metalloproteases (MMPs); signaling proteins.

Neuron	Astrocyte	Oligodendrocyte	Reactive Astrocytes
aggrecan	aggrecan	phosphacan	hyaluronan
brevican	brevican	versican	brevican
neurocan	neurocan	Tn-R	neurocan
phosphacan	phosphacan	MMPs	phosphacan
hyaluronan	hyaluronan		versican
Hapln1	versican		Tn-C
Hapln4	Tn-R		
Tn-R	Tn-C		
Tn-C	MMPs		
MMPs			
semaphorin 3A			

**Table 2:** Glycosaminoglycans (GAGs) composition. GAGs can contain galactose (Gal) or Uronic acid (glucuronic acid—GlcA or iduronic acid—IdoA) plus (+) Amino sugar (N-acetylgalactosamine—GalNAc and N-acetylglucosamine—GlcNAc. The various compositions of these units provide the disaccharide building components for GAGs (below).

Base Unit				
**Galactose (Gal) or**	**Uronic Acid**		**+ Amino sugar**	
	Glucuronic Acid (GlcA)	Induronic Acid (IdoA)	N-Acetylgalactoseamine (GalNAc)	N-Acetylglucosamine (GalNAc)
**Composition of Glycosaminoglycans (GAGs)**				
**Glycosaminoglycan**	**Repeated Units**			
Hyaluronan (HA)	GlcA		+ GlcNAc	
Chondroitin Sulphate	GlcA		+ GalNAc	
Heparan Sulphate	GlcA		+ GlcNAc	
Keratan Sulphate	Gal		+ GlcNAc	
Dermatan Sulphate	IdoA		+ GalNAc	

## Abbreviations

AMPARs	α-amino-3-hydroxy-5-methyl-4-isoxazolepropionic acid receptors
AD	Alzheimer’s disease
SHANK	SH3 and ankyrin repeat domains protein
ASD	Autism spectrum disorders
AQPs	Aquaporins
BBB	Blood brain barrier
BMECs	Brain microvascular endothelial cells
BDNF	Brain-derived neurotrophic factor
Bral1	Brain-specific hyaluronan-binding protein 1
Bral2	Brain-specific hyaluronan-binding protein 2
Crtl1	Cartilage-linking protein 1
VAMP3	Cellubrevin
CS	Chondroitin sulphate
CSPGs	Chondroitin sulphate proteoglycans
ChABC	Chondroitinase ABC
DAI	Diffuse axonal injury
GAG-GAG	Disaccharide polymers glucosaminoglycan- glucosaminoglycan
DLG4	Discs Large MAGUK scaffold protein 4
VAMP8	Endobrevin
ECM	Extracellular matrix
Gal	Galactose
GalNAc	Galactose-N-acetylogalactosamine
GABA	Gamma-aminobutyric acid
GCS	Glasgow Coma Scale
GPI	Glycosylphosphatidylinositol
GlcA	Glucosamine
GAGs	Glucosaminoglycans
GlcNAc	Glucose N-acetyloglucosamine
GlcA-GlcNAc	Glucuronic acid–acetylogalactosamine
GlcA	Glucuronic acid
GKAP	Guanylate kinase-associated protein
GK	Guanylate kinase-like domain
HOMER	Homer protein
HAPLN-1, -3 and -4	Hyaluronan and proteoglycan binding link protein
HAS	Hyaluronan synthase
HA	Hyaluronic acid (hyaluronan,
IdoA	Iduronic acid
MAGUK	Membrane-associated guanylate guanyl kinases
mGluR	Metabotropic glutamate receptor
MMPs	Metalloproteinases
VAMP5	Myobrevin
NRXN1	Neurexin 1
NRXN2	Neurexin 2
NRXN3	Neurexin 3
NLGN1	Neuroligin 1
NLGN3	Neuroligin 3
NLGN4X,	Neuroligin 4, X-linked precursor
NLGN4Y	Neuroligin 4, Y-linked precursor
NLGN	Neuroligin
NMDA(R)	N-methyl-D-aspartate receptors
PD	Parkinson’s disease
PNNs	Perineuronal nets
PSD	Postsynaptic densities
PSD95/DLG4	Postsynaptic density protein-95
PAZ	Presynaptic active zone
NRXN1, NRXN2, NRXN3	Neurexin 1,2,3
PGs	Proteoglycans
SNAP	Soluble N-ethylmaleimide sensitive factor attachment protein
SNARE	Soluble N-ethylmaleimide sensitive factor attachment protein receptor
SVs	Synaptic vesicles
VAMP1/VAMP2; VAMP1/2	Synaptobrevin ½
Syp	Synaptophysin
SNAP-25	Synaptosomal nerve-associated protein 25
v-SNAREs	Vesicle-associated SNARE proteins
Syt-1	Synaptotagmin 1
t-SNAREs	Target-localized SNARE proteins
Tn-C	Tenascin-C
Tn-R	Tenascin-R
TJs	Tight junctions
Ti-VAMP	Tetanus-Insensitive VAMP
TIMP	Tissue inhibitor of metalloproteinase
TBI	Traumatic brain injury
Trk(R)	Tropomyosin receptor kinase B
VAMP4	Vesicle-associated membrane protein 4
VAMP	Vesicle-associated membrane protein
VAMP7	Vesicle-associated membrane protein 7
